# Supplementary material for: MetaRibo-Seq measures translation in microbiomes
Source: Nat Commun. 2020 Jun 29;11:3268. doi: 10.1038/s41467-020-17081-z (PMC7324362; doi:10.1038/s41467-020-17081-z)
Supplement: Supplementary file 10 — Supplementary Data 7 [file 41467_2020_17081_MOESM10_ESM.zip › File2/Confidence_High_Taxonomy/290960_out.krona.html]

Javascript must be enabled to view this page.

members
magnitude
magnitudeUnassigned
count
unassigned
taxon
rank

290960\_out

173

173
2
superkingdom

phylum
1239
173

171
class
186801


SRS104036\_contig\_number\_contig-100\_16035.16036
order
186802
1
171

family
216572
158

459786
genus
158

4

SRS015579\_contig\_number\_19631SRS017701\_contig\_number\_contig-100\_21176.21176SRS053649\_contig\_number\_13344SRS1055067\_contig\_number\_contig-100\_3078.76631
1897011
species

1945593
species

SRS011134\_contig\_number\_36701SRS011302\_contig\_number\_27887SRS013098\_contig\_number\_4014SRS013382\_contig\_number\_995SRS013521\_contig\_number\_contig-100\_518.72994SRS013638\_contig\_number\_8312SRS013940\_contig\_number\_17691SRS013951\_contig\_number\_27674SRS014412\_contig\_number\_8023SRS014415\_contig\_number\_187SRS014459\_contig\_number\_40089SRS014683\_contig\_number\_18266SRS014855\_contig\_number\_1056SRS014923\_contig\_number\_40635SRS014948\_contig\_number\_14941SRS015065\_contig\_number\_15872SRS015190\_contig\_number\_contig-100\_1636.1636SRS015663\_contig\_number\_30734SRS015694\_contig\_number\_21196SRS015782\_contig\_number\_contig-100\_1947.223900SRS015890\_contig\_number\_24359SRS015960\_contig\_number\_contig-100\_1522.128259SRS016018\_contig\_number\_13045SRS016203\_contig\_number\_867SRS016753\_contig\_number\_5345SRS017103\_contig\_number\_33532SRS017191\_contig\_number\_18703SRS017821\_contig\_number\_20012SRS017916\_contig\_number\_11429SRS018351\_contig\_number\_1817SRS018541\_contig\_number\_12684SRS018836\_contig\_number\_16694SRS018888\_contig\_number\_7968SRS018984\_contig\_number\_26729SRS019030\_contig\_number\_16494SRS019178\_contig\_number\_contig-100\_2312.2313SRS019397\_contig\_number\_11228SRS019445\_contig\_number\_3418SRS019496\_contig\_number\_12998SRS019693\_contig\_number\_9076SRS019808\_contig\_number\_19962SRS020233\_contig\_number\_52365SRS020271\_contig\_number\_contig-100\_268.269SRS021484\_contig\_number\_14579SRS022137\_contig\_number\_13054SRS022609\_contig\_number\_24789SRS024435\_contig\_number\_34988SRS024625\_contig\_number\_contig-100\_5246.33202SRS024663\_contig\_number\_15092SRS043411\_contig\_number\_contig-100\_1889.100628SRS043768\_contig\_number\_18624SRS043841\_contig\_number\_8400SRS044535\_contig\_number\_15275SRS045004\_contig\_number\_23912SRS045713\_contig\_number\_10279SRS045739\_contig\_number\_5548SRS045826\_contig\_number\_1685SRS046502\_contig\_number\_12250SRS047014\_contig\_number\_contig-100\_6065.132576SRS047433\_contig\_number\_13949SRS048164\_contig\_number\_25046SRS049446\_contig\_number\_13016SRS049896\_contig\_number\_1857SRS049995\_contig\_number\_13077SRS050998\_contig\_number\_12407SRS051031\_contig\_number\_36554SRS053356\_contig\_number\_12432SRS053573\_contig\_number\_3047SRS054059\_contig\_number\_1964SRS054352\_contig\_number\_18493SRS054905\_contig\_number\_12024SRS055017\_contig\_number\_3244SRS055982\_contig\_number\_17311SRS058070\_contig\_number\_29404SRS062654\_contig\_number\_22050SRS062701\_contig\_number\_7443SRS063518\_contig\_number\_30920SRS063985\_contig\_number\_contig-100\_1288.129622SRS064276\_contig\_number\_16922SRS064757\_contig\_number\_22991SRS065397\_contig\_number\_4222SRS074964\_contig\_number\_1043SRS075078\_contig\_number\_30958SRS075341\_contig\_number\_2153SRS075773\_contig\_number\_20557SRS075821\_contig\_number\_19093SRS075984\_contig\_number\_15332SRS076756\_contig\_number\_7511SRS076804\_contig\_number\_4303SRS076929\_contig\_number\_contig-100\_281.152284SRS077024\_contig\_number\_4437SRS077127\_contig\_number\_15058SRS077231\_contig\_number\_11229SRS077294\_contig\_number\_13219SRS077552\_contig\_number\_25337SRS077641\_contig\_number\_18227SRS077991\_contig\_number\_contig-100\_4331.4332SRS078176\_contig\_number\_20672SRS078242\_contig\_number\_14910SRS097920\_contig\_number\_18385SRS098073\_contig\_number\_18692SRS098571\_contig\_number\_65230SRS098644\_contig\_number\_29277SRS098717\_contig\_number\_21180SRS098827\_contig\_number\_11548SRS098881\_contig\_number\_3980SRS100907\_contig\_number\_8742SRS101376\_contig\_number\_25027SRS103987\_contig\_number\_5648SRS104084\_contig\_number\_8323SRS1041037\_contig\_number\_contig-100\_371.61232SRS1041092\_contig\_number\_contig-100\_422.89080SRS1041116\_contig\_number\_855SRS1041134\_contig\_number\_5806SRS1041142\_contig\_number\_7920SRS104311\_contig\_number\_21458SRS104400\_contig\_number\_965SRS104912\_contig\_number\_4724SRS104975\_contig\_number\_21296SRS1055022\_contig\_number\_20826SRS1055099\_contig\_number\_contig-100\_288.57535SRS142503\_contig\_number\_3481SRS142542\_contig\_number\_12163SRS142599\_contig\_number\_17200SRS142923\_contig\_number\_17910SRS143070\_contig\_number\_6728SRS143085\_contig\_number\_contig-100\_130.49183SRS143148\_contig\_number\_26477SRS143181\_contig\_number\_2094SRS143342\_contig\_number\_12722SRS143523\_contig\_number\_7739SRS143598\_contig\_number\_19414SRS143895\_contig\_number\_29222SRS143991\_contig\_number\_21152SRS144362\_contig\_number\_18988SRS144506\_contig\_number\_5599SRS144603\_contig\_number\_13641SRS145497\_contig\_number\_18725SRS147271\_contig\_number\_29795SRS147346\_contig\_number\_67833SRS148159\_contig\_number\_7486SRS148253\_contig\_number\_12540SRS148319\_contig\_number\_12720SRS148424\_contig\_number\_27664SRS148784\_contig\_number\_6021SRS148817\_contig\_number\_12273SRS149325\_contig\_number\_contig-100\_199.102934SRS150029\_contig\_number\_11708SRS893295\_contig\_number\_10326SRS893300\_contig\_number\_12330SRS893373\_contig\_number\_1745SRS893378\_contig\_number\_14078SRS893383\_contig\_number\_contig-100\_11034.144655SRS971275\_contig\_number\_23492
154

1

SRS143417\_contig\_number\_23538
1898207
species

9
family
541000

species
2292180

SRS013687\_contig\_number\_contig-100\_569.121282SRS014235\_contig\_number\_contig-100\_2579.246168SRS022524\_contig\_number\_12537SRS024075\_contig\_number\_6376SRS050925\_contig\_number\_29472SRS104327\_contig\_number\_6415SRS893231\_contig\_number\_5829SRS893279\_contig\_number\_13974
8


SRS056273\_contig\_number\_14596
species
2292270
1

1

SRS101433\_contig\_number\_9178
1950935
species

1
172733
species

SRS019068\_contig\_number\_106970

1

SRS148721\_contig\_number\_39273
1262992
species

1

SRS074670\_contig\_number\_8421
species
1897028
